# Age Demographics of Subjects Enrolled in Global, Interventional Phase 3 Melanoma Clinical Trials

**DOI:** 10.1007/s43441-021-00362-0

**Published:** 2022-01-09

**Authors:** Reema Shah, Nil Patel, Yasha Patel, Michael Toscani, Joseph Barone, Paul F. Weber

**Affiliations:** 1 Ernest Mario School of Pharmacy at Rutgers, The State University of New Jersey, Piscataway, NJ USA; 2 Rutgers Robert Wood Johnson Medical School, New Brunswick, NJ USA

**Keywords:** Melanoma, Trial participants, Age demographics, Clinical trial design, Geriatric oncology

## Abstract

**Background:**

Melanoma is a skin cancer with a rising worldwide incidence of just over 280,000 individuals with the greatest burden of illness in European, New Zealander, and Australian populations. Patients are diagnosed with melanoma with the mean and median ages being 65 and 59 years old, respectively. Phase 3 trials not only provide a wide representation of the target population but also study the efficacy for a certain intervention.

**Objective:**

The objective of this literature review is to analyze patient demographics of phase 3 trials for melanoma and identify if there is a true disparity between the clinical trial age demographics and the natural epidemiological age demographics.

**Data Sources:**

The authors conducted a search on clinicaltrials.gov, a publicly available resource that lists clinical trials and their data. The reported mean and median ages for each trial were extracted after determining if each trial meets our inclusion criteria. Weighted mean and median ages were calculated using an online calculator.

**Data Summary:**

Data from 35 trials were evaluated with 30 trials reporting a weighted mean age of 55.85 years and 5 trials reporting a weighted median age of 55.14 years.

**Conclusion:**

Based on the results, melanoma clinical trials enroll patients who are younger than the epidemiological mean and median ages. Due to this underrepresentation of the elderly patients with melanoma, clinical trials may provide limited application for the use of their results.

## Introduction

Melanoma is one of the most serious forms of skin cancer and its incidence is high not only in the young population but also among the geriatric populations [1, 2]. It is considered to be a heterogeneous disease with a complex etiology [3]. While it only encompasses 1% of all skin cancers, it is responsible for the largest amount of skin cancer deaths [3]. It develops when normal, healthy color-producing cells grow out of control due to their inability to halt their own growth.

As of 2017, the prevalence of patients diagnosed with melanoma in the USA was approximately 1.2 million cases, making up about 5.6% of all new cancer cases in the USA [4]. The incidence estimates for melanoma for 2021 is 106,110 new melanoma cases to be diagnosed and about 7180 patients expected to die of melanoma in the USA alone [5]. The worldwide incidence of melanoma has been rising over the past 50 years and is approximately 280,000 individuals globally [1]. While the rates for melanoma have increased in the past few decades, it is mostly varied by age [5]. The risk of melanoma increases with age, and the mean and median age of diagnosis in the USA is 65 and 59 years, respectively [5, 6]. While the risk of melanoma increases with age, it can occur in individuals who are younger than the age of 30 [5]. It is more common in males, but the rates are higher in females before the age of 50 [5]. The incidence of disease in the USA is higher in non-Hispanic White populations (21.6/100,000) compared to that of Hispanic and Black populations being 4.5/100,000 and 1.0/100,000, respectively [7]. European, New Zealander, and Australian populations represent a higher burden of illness compared to the rest of the world because they include predominantly fair-skinned individuals as well as an emphasis on tanning [1, 8].

Melanoma occurs when melanocytes or pigmented cells become cancerous. Melanocytes produce melanin and are found at the bottom of the epidermis. An easy way to differentiate abnormal vs normal skin cells is via the “ABCDE” rule: asymmetry, border irregularity, color, diameter, and evolving. Asymmetry is when half of the lesion does not match. Border irregularity is when the edges are ragged, notched, or blurry. The color of the lesions can be shades of brown, black, or tan; sometimes areas of white, gray, red or blue may be present. The width or diameter of the lesion is greater than ¼ inch, about the size of a pencil eraser; it can also be smaller when first detected. It is also important to notice if the lesion has been evolving or changing in shape, size, color, or appearance. While melanomas don’t cause pain, they can ooze, itch, or bleed; however, bleeding is usually a sign of an advanced melanoma [9, 10].

There are various types of treatments for melanoma, but it depends on the primary location of the melanoma, the size, the genetic changes, the rate of the growth, and other health conditions of the patients. Typically, surgery or excision is the main course of treatment, but it depends on the location of the melanoma tumor. It is performed if the melanoma is local or regional and sometimes if it is metastatic. Depending on the symptoms of the individual and the location of the melanoma, radiation therapy may be given to prevent the recurrence of the cancer [10].

Systemic therapies, which include immunotherapy, targeted therapy, and chemotherapy, can be administered to patients. Immunotherapy treating melanoma is comprised of programmed cell death protein 1 (PD-1) inhibitor monoclonal antibodies (e.g., nivolumab and pembrolizumab), programmed death-ligand (PD-L1) inhibitor (e.g., atezolizumab), cytotoxic T-lymphocyte associated molecule-4 (CTLA-4) inhibitor (e.g., ipilimumab), a combination of ipilimumab and nivolumab, interleukin-2 (IL-2), virus therapy (e.g., talimogene laherparepvec), and interferon (e.g., high-dose interferon alfa-2b and pegylated interferon alfa-2b). Targeted therapy consists of BRAF inhibitors (e.g., dabrafenib), MEK inhibitors (e.g., trametinib), combination of BRAF and MEK inhibitors, BCR-ABL tyrosine kinase inhibitors (e.g., imatinib), or tumor agnostic treatment (e.g., larotrectinib). Immunotherapy and targeted therapy are more commonly used than chemotherapy due to their effectiveness and safety profile. Some chemotherapy drugs utilized include dacarbazine for melanoma and temozolomide for stage IV melanoma. Isolated limb infusion therapy can be utilized if the melanoma has spread and appears to have multiple tumors in the arm or legs [10].

Previous studies have demonstrated there is a lack of inclusivity of elderly patients in clinical trials. Ramamoorthy et al. studied 12 new molecular entity (NME) applications in clinical trials approved by the FDA between January 2008 and February 2013 in select oncology products: breast, prostate, lung, and colorectal. Select oncology products approved between 2008 and 2013, only 41% of the 22,481 participants were ≥ 65 years of age. Between 2014 and 2017, 39% of the 3612 participants were ≥ 65 years of age [11]. Borad et al. reported younger patients being enrolled more than the true demographic of multiple myeloma in phase 3 clinical trials [12]. Randomized clinical trials observing drug interventions for ischemic heart disease starting from 2006 and after to measure the exclusion of the elderly population were examined. The overall mean of the participants in the trial was 62.7 years and of the 839 trials, 53% failed to include the elderly [13]. Even though the risk of melanoma increases with age, there are no studies that address the disparity between participants in clinical trials and their age. This is paramount to study design and provides a better representation of how to improve patient outcomes when considering different types of therapies that are available especially for geriatrics.

The purpose of this literature review is to determine patient age for phase 3 global, interventional clinical trials for melanoma compared to the natural history of the disease. By studying phase 3 trials, it provides a larger representation of the population for the purpose of efficacy. Through literature searches, the goal is to determine if there is an unmet need for research in this disease state for the elderly populations.

## Methods

A search of melanoma on clinicaltrials.gov, a public resource listing information on clinical trials, was conducted. The search term “melanoma” was inputted to specify the disease state, as well as two filters, “with results” and “phase 3”, to target all phase 3 melanoma trials with results available. The inclusion criteria for this analysis included trials that treated only melanoma, phase 3 trials, trials with results available on clinicaltrials.gov, and trials that evaluated a pharmacological or procedural intervention to treat melanoma. The exclusion criteria for this review included trials that treat more than one type of malignancy, trials that did not study melanoma, trials that investigated supportive care for melanoma, and trials that did not report a mean or median age. Two authors independently screened each trial result for eligibility, each creating a list of trials ineligible for this review. The lists were compared and overlapping trials were excluded from this study.

For each trial that was included in this review, data was manually taken from clinicaltrials.gov using the baseline characteristics section within the study results tab. If the website did not state the mean/median age, the publication linked to the study was reviewed to see if the mean/median age was stated, and if not, the trial was excluded from this review. All the studies included either had a mean or median age, but not both, so extracting both the mean and median ages from a single trial was not necessary. The authors retrieved mean or median age, number of participants in the study, studied pharmacological or procedural interventions, and dates of trials.

After collecting these values, a weighted average for the mean and median was conducted to account for the different number of participants per trial. These numbers were compared to the epidemiological mean and median age for patients diagnosed with melanoma.

## Results

**Fig. 1:**
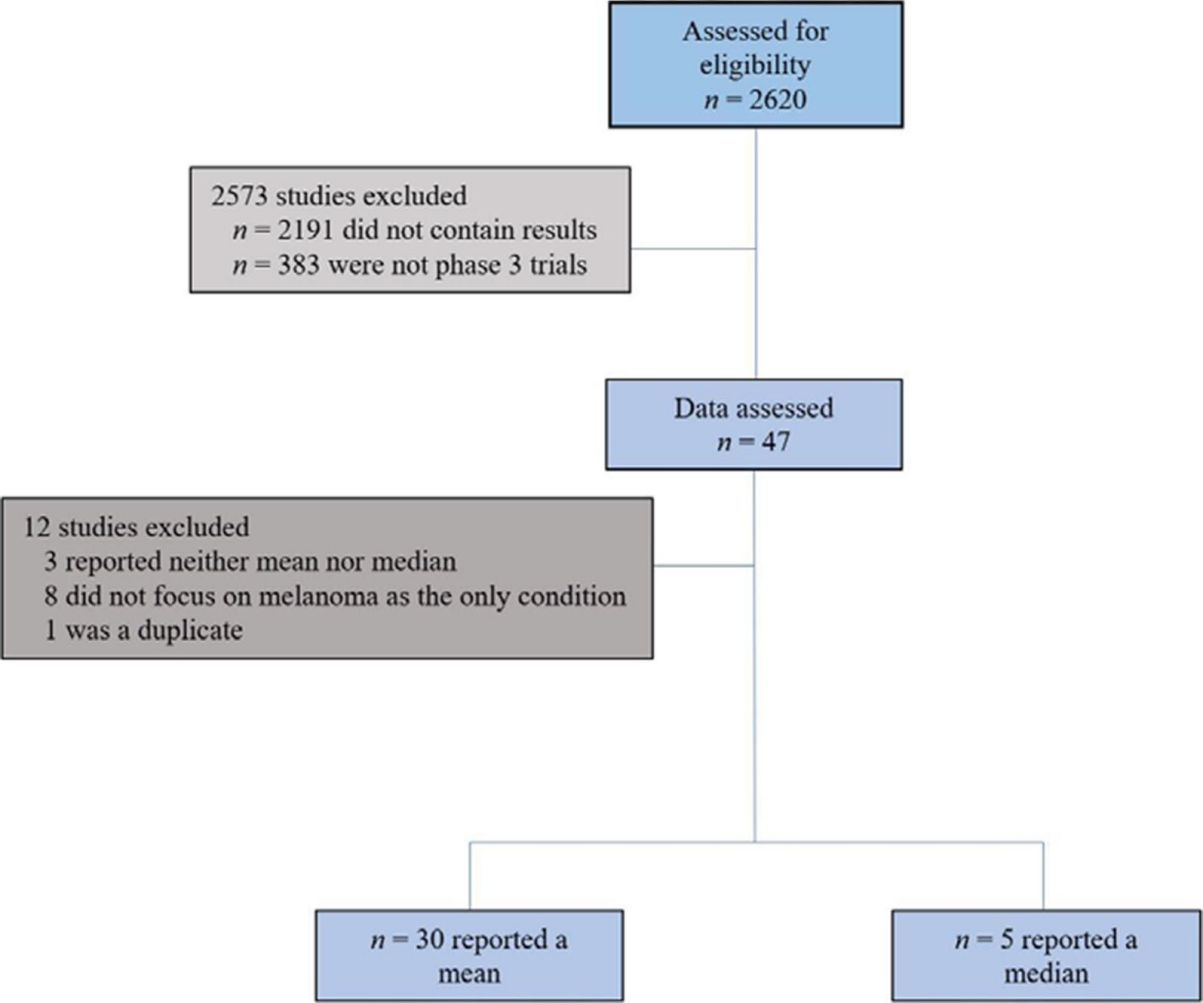
Flowchart of the trials from clinicaltrials.gov that were included in the review

A clinicaltrials.gov search returned 2620 trials (Fig. 1) on melanoma. Of these trials, 2573 trials were excluded because they did not contain results (2191 trials) or were not phase 3 clinical trials (382 trials). The remaining 47 trials were assessed for eligibility in this study. Of the 47 trials, five were excluded because they did not report a mean or median age, one was a duplicate of an eligible trial, and six were excluded because they treated more than one type of cancer. The remaining 35 trials that were global phase 3, with results, and treating melanoma were included in this study. The 35 trials spanned from December 1998 to December 2020 and studied a total of 20,912 patients. Of the 35 trials, 30 reported a mean age and five reported a median age; none of the trials reported both a mean and median age.

**Fig. 2:**
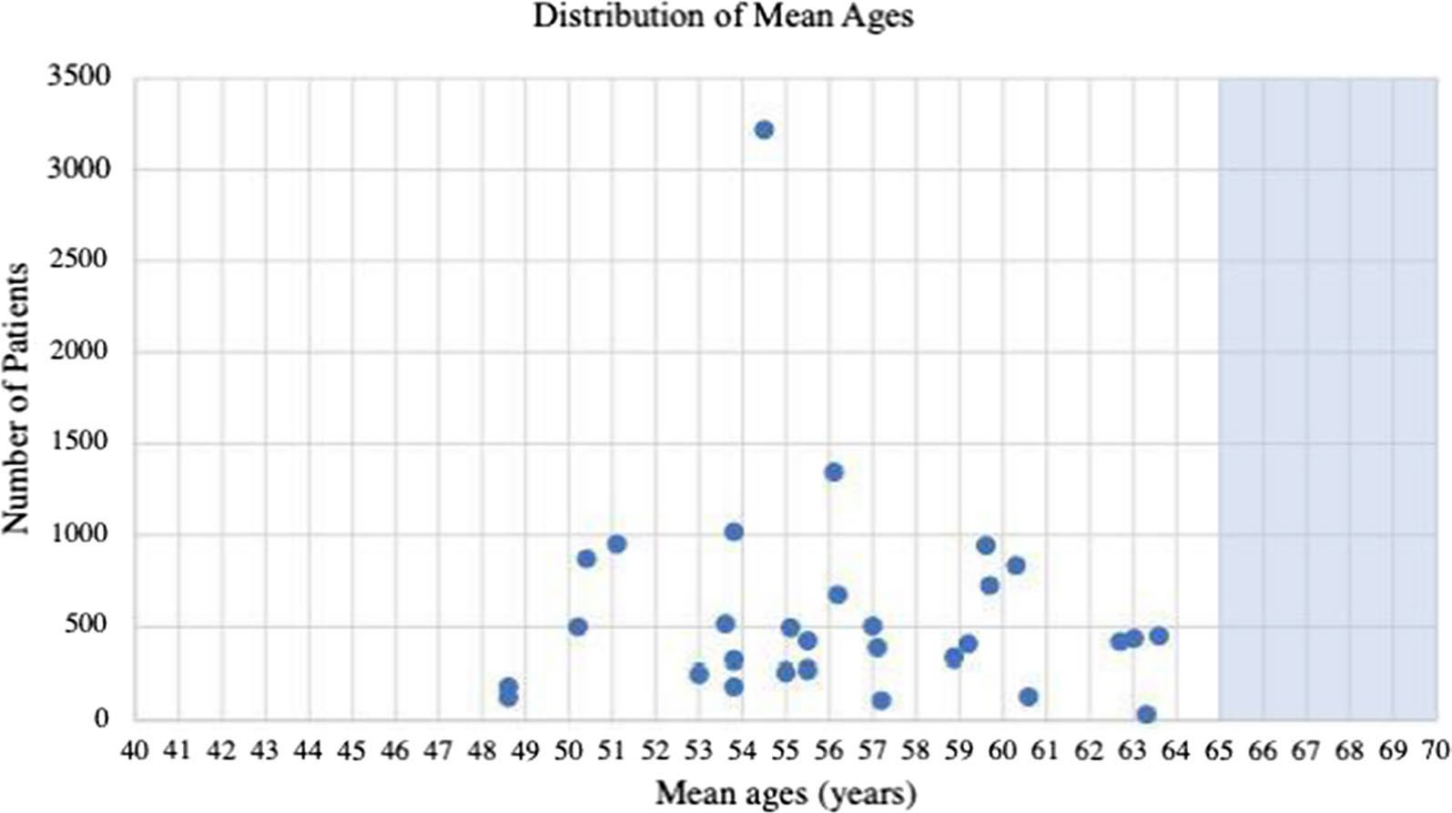
Mean ages of participants reported from 30 clinical trials. Highlighted area shows the epidemiological mean of diagnosis of melanoma

**Fig. 3:**
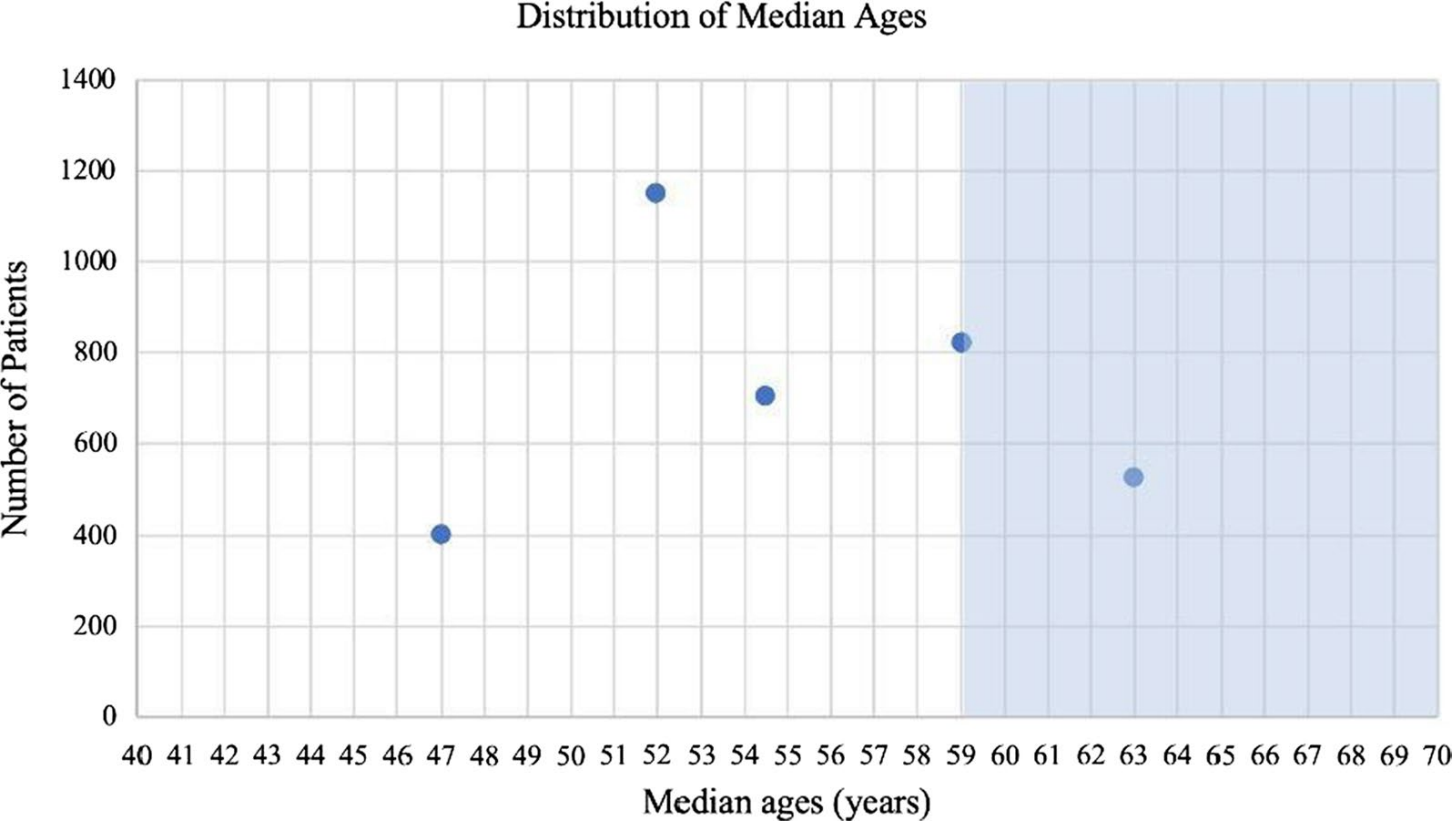
Median ages of participants reported from 30 clinical trials. Highlighted area shows the epidemiological median of diagnosis of melanoma

A weighted average to find the average mean and median ages for all the trials was utilized to account for the different number of participants per trial. The weighted mean age of the 30 trials was found to be 55.85 years and the weighted median age of the five trials was found to be 55.14 years (Table 1, Figs. 2, 3). The average number of participants in the trials that reported a mean was 576.8 participants and the average number of participants in the trials that reported a median was 721.6 participants.

**Table 1:** Average mean and median ages of melanoma from clinical trials compared to that of epidemiologic mean and median ages of patients

Type of age represented in trial	Clinical trials average (years)	Epidemiologic age (years) [5, 6]	Difference (years)
Mean age	55.85	65	9.15
Median age	55.14	59	3.86

Staging was a factor for representation of elderly patients. Out of the 35 trials analyzed in this manuscript, 20.0% (7/35) included stage III melanoma, 8.6% (3/35) included stage IV melanoma, 62.9% (22/35) included both Stage III and IV, and 8.6% included stages 0–II. Most of the trials selected patients who were in Stage III (unresectable) and/or IV, regardless of demographics. There was no direct correlation between lower mean age and lower stage.

Several pharmaceutical interventions were used throughout the clinical trials that were included in this study. A majority of compounds were biologics, including monoclonal antibodies such as anti-PD-1 inhibitors pembrolizumab and nivolumab, the anti-PDL-1 inhibitor atezolizumab, the anti-CTLA-4 inhibitor ipilimumab, and other biologics including filgrastim, interleukin-2, talimogene laherparepvec among others. Targeted therapy was studied in several of the trials, with drugs such as the MEK inhibitor cobimetinib and two BRAF inhibitors, vemurafenib and trametinib. A select number of trials studied chemotherapeutic options such as the taxane paclitaxel, a vinca alkaloid vinblastine, and the platinum agents carboplatin and cisplatin. Radiation was also used in some trials in combination with the pharmaceutical agents. None of the trials studied procedural therapies such as allogeneic stem cell transplants.

## Discussion

**Fig. 4:**
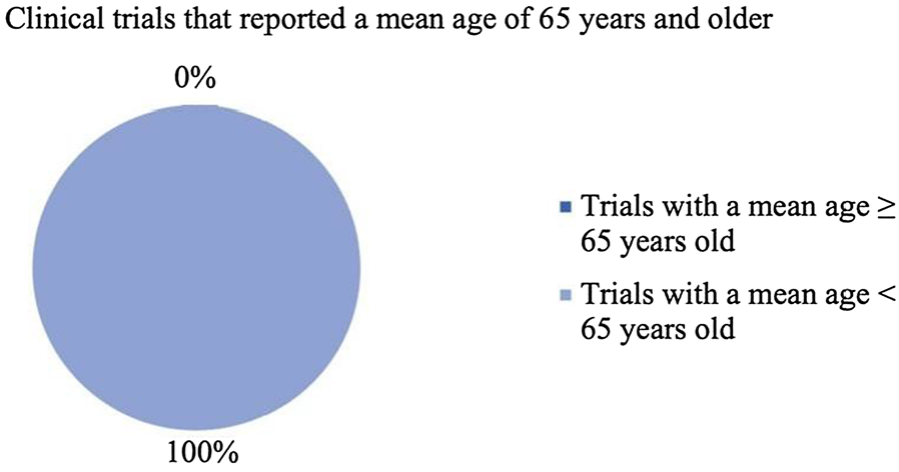
Percentage of clinical trials with a mean age of participants greater than 65 years old

**Fig. 5:**
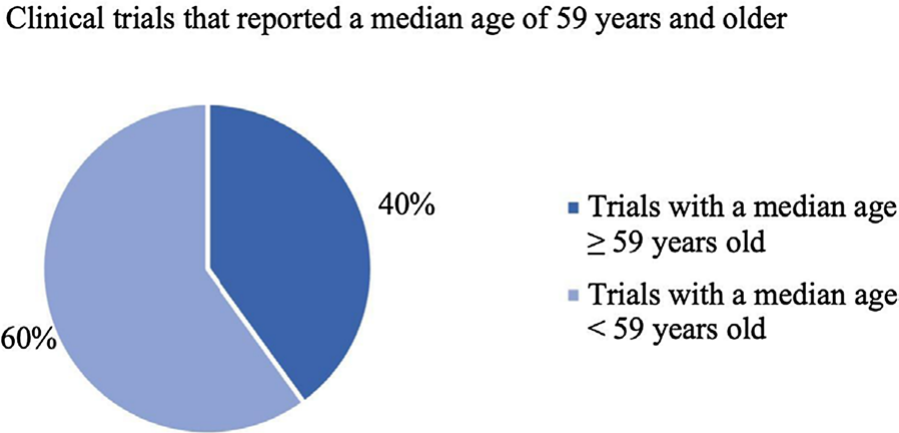
Percentage of clinical trials with a median age of participants greater than 59 years old

Based on the results of this literature review, it has been determined that there may be an age disparity between the epidemiological mean and median ages for patients diagnosed with melanoma and the weighted mean and median ages of the patients participating in phase 3 (including pivotal) clinical trials treating melanoma. The difference between these two values was found to be 9.15 and 3.86 years for the mean and median differences, respectively. Furthermore, none of the trials that reported a mean age had a mean age that was at least the epidemiological mean age, which is 65 years (Fig. 4). Two out of the five trials that contained the median age of patients had at least the epidemiologic median age, which is 59 years (Fig. 5). This led to the conclusion that patients included in the clinical trials represent a younger population than the average melanoma population.

The geriatric population represented in these trials were defined in the respective label section (Section 8.5) for the drugs that were studied and approved for any melanoma indication. According to the package inserts for nivolumab and ipilimumab, the percentage of the population in the CHECKMATE-067 trial studying nivolumab versus nivolumab plus ipilimumab that were or geriatric age greater than 65 years old was 41% [14, 15] According to the package insert for pembrolizumab, 48% of the subjects were enrolled in trials for melanoma, non-small cell lung cancer, head and neck squamous cell carcinoma, or urothelial carcinoma; however, this is a combination of four tumor types, so this number may not accurately represent the percentage of individuals over 65 for melanoma. Keytruda [16] Lastly, according to the package inserts of trametinib, 21% of the subjects were enrolled in the COMBI-d, COMBI-v, and COMBI-AD trials, which studied trametinib in various combinations with dabrafenib and vemurafenib for the treatment of melanoma, were older than 65 [17].

Several reasons are possible for this age disparity. Elderly patients often have an increased number of comorbidities compared to younger patients, thus negatively affecting the therapy’s efficacy. This increased number of comorbidities that elderly patients deal with leads them to suffer more serious adverse events, limiting their participation in these trials. As a result, the melanoma trials may have recruited younger patients to positively affect their therapy’s therapeutic profile. Furthermore, recruiting elderly patients into clinical trials may have been more difficult for them due to unstable medical conditions, frequent hospitalizations, and unwillingness to travel due to transportation concerns [18]. Elderly patients can also have altered pharmacokinetics and pharmacodynamic parameters compared to younger individuals. This can influence the safety and therapeutic response of the intervention being studied. According to De Luca et al., there is lack of evidence for the efficacy, safety, and tolerability in the elderly population with melanoma. Studies fail to include patients that are greater than the age of 75 years old [19]. If the elderly patients are included, they do not accurately demonstrate the older population with cancer [20]. As a result, these trials lack a fair representation of the epidemiologic mean and median ages for melanoma. Health care professionals in their own practice who use the therapy being studied can possibly endanger the patient because the clinical trial age group may not represent their patient’s age. The clinical trial team needs to increase its efforts to recruit more elderly patients who represent the epidemiological age more closely, misrepresentation of the population demographic age would likely falsely elevate any therapy’s efficacy and may not translate into the real world.

There are several limitations to this literature review, so our findings should be interpreted carefully. Only 35 trials were included in this review, with only five of them reporting a median age. A small sample size cannot fully reflect all of the clinical trials on melanoma, as there are many ongoing with results still waiting to be published. These ongoing trials may or may not contain elderly participants as the information was not accessible to the authors of this review, thus not stated. Furthermore, this review focused on phase 3 melanoma clinical trials that were listed on clinicaltrials.gov that had certain outcome descriptions, not phase 1 or 2 nor any post-marketing studies reflecting real world data. As a result, our findings may not represent all the melanoma trials that have been conducted.

In March 2020, the FDA issued a draft guidance that recommended the inclusion of older adults (age greater than 65) in clinical trials that studied drugs to treat cancer. In an aging population, the inclusion of the elderly in clinical trials is essential to represent the overall target population for the disease state. By studying the target population that health care professionals are treating, the drugs being studied in clinical trials can be better represented by the correct age group to evaluate the benefit-risk profile accurately and reduce the risk of safety concerns, ineffectiveness, and wasted resources. The recommendations the FDA made in the draft guidance reflect the results in this literature review [21].

## Conclusion

The results of this literature review may demonstrate that patients in phase 3 melanoma clinical trials do not represent the true demographic melanoma patient age. This disparity may be a disadvantage to health care professionals treating melanoma due to the inability to generalize the clinical trial results to their patients, particularly the elderly. As the population continues to age and more oncologic agents are introduced into the market, this issue becomes even more pertinent. Moving forward, future clinical trials should confirm these preliminary findings with elderly patients to see if age plays a significant influence on the safety and efficacy of therapies.

## References

[1] Raimondi S, Suppa M, Gandini S (2020). Melanoma epidemiology and sun exposure. Acta Derm Venereol.

[2] American Society of Clinical Oncology. Melanoma: introduction. https://www.cancer.net/cancer-types/melanoma/introduction. Accessed 5 Jan 2021.

[3] Nikolaou VA, Stratigos AJ, Flaherty KT (2012). Melanoma: new insights and new therapies. J Invest Dermatol.

[4] National Cancer Institute. Cancer stat facts: melanoma. https://seer.cancer.gov/statfacts/html/melan.html. Accessed 5 Jan 2021.

[5] American Cancer Society. Melanoma. https://www.cancer.org/cancer/melanoma-skin-cancer/about/key-statistics.html. Accessed 28 Feb 2021.

[6] AIM at Melanoma Foundation. Is age a risk factor for melanoma? https://www.aimatmelanoma.org/melanoma-101/understanding-melanoma/melanoma-risk-factors/age-and-risk. Accessed 5 Jan 2021.

[7] Erdei E, Torres SM (2010). A new understanding in the epidemiology of melanoma. Expert Rev Anticancer Ther.

[8] Karimkhani C, Green AC, Nijsten T (2017). The global burden of melanoma: results from the global burden of diseases Study 2015. Br J Dermatol.

[9] National Comprehensive Cancer Network. Melanoma. (Version 1. 2018), https://www.nccn.org/patients/guidelines/content/PDF/melanoma-patient.pdf. Accessed 5 Jan 2021.

[10] American Society of Clinical Oncology. Melanoma: signs and symptoms. https://www.cancer.net/cancer-types/melanoma/symptoms-and-signs. Accessed 5 Jan 2021.

[11] Ramamoorthy A, Knepper TC, Merenda C (2018). Demographic composition of select oncologic new molecular entities approved by the FDA between 2008 and 2017. Clin Pharmacol Ther.

[12] Borad A, Saeed H, Toscani M (2020). Age demographics of subjects enrolled in interventional phase 3 multiple myeloma clinical trials. J Oncol Pharm Pract.

[13] Bourgeois FT, Orenstein L, Ballakur S (2017). Exclusion of elderly people from randomized clinical trials of drugs for ischemic heart disease. J Am Geriatr Soc.

[14] Opdivo (nivolumab) package insert. https://www.accessdata.fda.gov/drugsatfda_docs/label/2021/125554s090lbl.pdf. Accessed 17 Oct 2021.

[15] Yervoy (ipilimumab) package insert. https://www.accessdata.fda.gov/drugsatfda_docs/label/2015/125377s073lbl.pdf. Accessed 17 Oct 2021.

[16] Keytruda (pembrolizumab) package insert. https://www.accessdata.fda.gov/drugsatfda_docs/label/2021/125514s096lbl.pdf. Accessed 17 Oct 2021.

[17] Mekinist (trametinib) package insert. https://www.accessdata.fda.gov/drugsatfda_docs/label/2014/204114s001lbl.pdf. Accessed 17 Oct 2021.

[18] Macias FM, Ramsay RE, Rowan AJ (2007). Recruitment and retention in clinical trials of the elderly. Int Rev Neurobiol.

[19] De Luca R, Meraviglia S, Blasi L (2020). Nivolumab in metastatic melanoma: good efficacy and tolerability in elderly patients. Curr Oncol (Toronto, Ont.).

[20] Schuurman MS, Hollestein LM, Bastiaannet E (2020). Melanoma in older patients: declining gap in survival between younger and older patients with melanoma. Acta Oncol.

[21] U.S. Food and Drug Administration. Inclusion of older adults in cancer clinical trials. https://www.fda.gov/regulatory-information/search-fda-guidance-documents/inclusion-older-adults-cancer-clinical-trials. Accessed 9 Jan 2021.

